# Oxidative Stress in the Carcinogenicity of Chemical Carcinogens

**DOI:** 10.3390/cancers5041332

**Published:** 2013-10-28

**Authors:** Anna Kakehashi, Min Wei, Shoji Fukushima, Hideki Wanibuchi

**Affiliations:** 1 Department of Pathology, Osaka City University Graduate School of Medicine, 1-4-3 Asahi-machi, Abeno-Ku, Osaka 545-8585, Japan; 2 Japan Bioassay Research Center, Japan Industrial Safety and Health Association, 2445 Hirasawa, Hadano, Kanagawa 257-0015, Japan

**Keywords:** oxidative stress, carcinogenicity, chemical carcinogen, low dose

## Abstract

This review highlights several *in vivo* studies utilizing non-genotoxic and genotoxic chemical carcinogens, and the mechanisms of their high and low dose carcinogenicities with respect to formation of oxidative stress. Here, we survey the examples and discuss possible mechanisms of hormetic effects with cytochrome P_450_ inducers, such as phenobarbital, α-benzene hexachloride and 1,1-bis(*p*-chlorophenyl)-2,2,2-trichloroethane. Epigenetic processes differentially can be affected by agents that impinge on oxidative DNA damage, repair, apoptosis, cell proliferation, intracellular communication and cell signaling. Non-genotoxic carcinogens may target nuclear receptors and induce post-translational modifications at the protein level, thereby impacting on the stability or activity of key regulatory proteins, including oncoproteins and tumor suppressor proteins. We further discuss role of oxidative stress focusing on the low dose carcinogenicities of several genotoxic carcinogens such as a hepatocarcinogen contained in seared fish and meat, 2-amino-3,8-dimethylimidazo[4,5-*f*]quinoxaline, arsenic and its metabolites, and the kidney carcinogen potassium bromate.

## 1. Introduction

In aerobic organisms, the balance in production of various oxidants like reactive oxygen and nitrogen species (ROS/RNS), which are invariable components of the aerobic metabolism, and their elimination by intracellular antioxidants of enzymatic nature such as superoxide dismutase (SOD), catalase and glutathione peroxidase (GTPx) and nonenzymatic nature such as radical scavengers like vitamin E, ferritins, different thiols, and others helps to maintain cellular homeostasis and to protect from formation of cellular oxidative stress [1,2,3]. On the other hand, organisms are exposed daily to oxidants generated either endogenously or exogenously (water and air pollutants, cigarette smoke, *etc.*), which physical and chemical characteristics (size, transition metal content, speciation, stable free radicals, *etc.*) play an important role in formation of oxidative stress. Continuous cellular damage induced by ROS/RNS is known to result in sustained cell proliferation, which if occurs in an environment rich in inflammatory cells, growth factors and activated stroma, will potentiate carcinogenesis [1]. Furthermore, oxidative stress itself initiates the synthesis of mediators of inflammation in cells and initiation of carcinogenic mechanisms [2,3].

Carcinogenesis is known to be promoted by: (1) direct induction of oxidative stress due to irradiation DNA damage produced by ROS [4]; and (2) indirect actions with possible consequences for homeostasis and reactions, involving inflammation following enhanced nitric oxide (NO) and RNS production, which triggers a cascade of oncogenic events [1]. It is necessary to note that chronic infection and inflammation have become well recognized as risk factors for a variety of human cancers. It has been proposed that ROS/RNS, both formed in infected and inflamed tissues, play roles in the multistage carcinogenic process. NO, a potential toxic substance with free radical properties, is generated from L-arginine by constitutive NO synthase or iNOS. Furthermore, one of the possible mechanisms underlying enhancement of initiation and promotion in carcinogenesis by oxidative stress is the ability to induce prostaglandin formation catalyzed by the enzyme cyclooxygenase (Cox) which converts arachidonic acid to prostaglandins. Presently, two Cox enzymes, Cox1 and Cox2, an important mediators of inflammation, whose synthesis can be stimulated rapidly by tumor promoters, are known [5]. 

ROS are genotoxic in principle, and the question arises as to whether chemicals that increase ROS production will add to an endogenously produced background level of DNA lesions, or whether compensatory mechanisms that may result in non-linear dose-effects exist. Endogenous ROS cause detectable background levels of DNA damage, namely in the form of oxidized bases, apurinic (AP) sites, and strand breaks. Oxygen radicals also attack other cellular components such as lipids, to generate reactive intermediates that couple to DNA and give rise to exocyclic etheno- and propane-adducts and 1,*N*^6^-ethenodeoxyguanosine and 3,*N*^4^-ethenodeoxycytidine [6,7,8]. Such adducts will have mutation-associated consequences upon cell replication [9]. The continuous production of free radicals from chemicals has stimulated organisms to evolve repair systems for oxidative base modifications or chromosome breaks. Alteration to DNA molecules triggers repair, and frequent activation may increase the general repair capacity, irrespective of the cause of the damage. Repeated exposure to ROS may thus lead to an adaptive response, mitigating the mutagenicity of oxidative DNA lesions. DNA repair is a crucial factor in maintaining a low steady-state level of DNA damage and its impairment is implicated in processes that promote human cancer [10].

## 2. Induction of Oxidative Stress by Environmental Chemical Carcinogens and Toxicants

Functions of three “orphan” nuclear receptor superfamily members, designated constitutive androstane receptor (CAR; NR1I4), pregnane X receptor (PXR; NR1I2) and peroxisome proliferator receptors (PPARs), in respectively mediating the induction of hepatic cytochrome P_450_ isoenzymes (CYPs) belonging to families CYP2, CYP3, and CYP4 and other proteins involved in the detoxification and elimination of chemical carcinogens from the body are well recognized [11,12,13,14,15,16]. CAR and PXR are expressed in the liver, intestine, lung, and other tissues, where they have central roles in detecting and removal of xenobiotics [12,17]. CAR is activated by a restricted set of chemical carcinogens like phenobarbital (PB) and the planar hydrocarbon 1,4-bis[2-(3,5-dichloropyridyloxy)]-benzene (TCPOBOP) [15,18]. CAR was originally demonstrated to regulate *CYP2B* expression and like PXR, to bind to xenobiotic response elements as a heterodimer with 9-*cis-*retinoic acid receptor [16]. PPARs activation has been linked with liver tumor formation and the mechanisms include induction of sustained oxidative stress, enhanced cell replication, promotion of preneoplastic lesions, and inhibition of apoptosis [19]. In particular, the activation of PPARα by peroxisome proliferators in rats and mice was reported to produce oxidative stress, due to the induction of enzymes like CYP4A1 and fatty acyl coenzyme A (CoA) oxidase (AOX) [20]. Recently, the existence of a cross talk between these receptors providing a mechanism for amplifying the body’s detoxification response to a broad range of chemicals was reported [21,22,23].

ROS are generated due to induction of various cytochrome P_450_ isoenzymes during detoxification of chemical carcinogens, lipid peroxidation and other intracellular processes and involved in regulation of numerous factors, such as transcription factors kappa B, NFkB and AP-1, partly through protein kinase C activation [24,25] and PPARγ leading to chronic inflammation [26]. Furthermore, the transcription factor NF-E2-related factor 2 (Nrf2) is considered responsible for regulating a battery of antioxidant and cellular protective genes, primarily in response to oxidative stress [27]. ROS oxidize lipid and protein molecules, generating intermediates, which can react with DNA and form adducts, or directly attack the DNA, damaging the bases and deoxyribose residues and producing single strand breaks.

8-Hydroxy-2'-deoxyguanosine (8-OHdG), a well-known oxidative DNA damage marker, was reported to be involved in chemical carcinogenesis in various experimental models [28]. Induction of a significant and steady elevation of 8-OHdG is thought to play an integral role in chemical carcinogenesis [29]. It has been shown to cause mutations, predominantly G to T transversions [30]. The time- and dose-dependent generation of 8-OHdG in rat hepatic DNA have been demonstrated after single i.p. administration of genotoxic carcinogens including diethylnitrosamine (DEN) and aflatoxin B1 [28,31,32]. In addition, 8-OHdG levels assessed after 1 week of dietary MeIQx application were also dose-dependently increased, although the effect was not observed at the end of the treatment period [33]. These effects are suggested to be involved in initiation of hepatocarcinogenesis by genotoxic chemicals. Previously, in case of non-genotoxic chemical carcinogens, it has been concluded that only long-term high dose treatment results in accumulation of 8-OHdG [34]. However, recent studies demonstrated its dose and time-dependent generation after 1 week of administration of such non-genotoxic carcinogens as PB and ethyl *tert*-butyl ether (ETBE) [35,36]. Since 8-OHdG in DNA is attributed to production of ROS, especially hydroxyl radicals [37] and can be rapidly repaired [38], the actual level in the tissue is determined by changes in rates of these processes. In addition, it is known that glutathione (GSH) deficiency, or a decrease in the GSH/glutathione disulphide (GSSG) ratio, leads to an increased susceptibility to oxidative stress implicated in the progression of cancer, elevated GSH levels increase the antioxidant capacity and the resistance to oxidative stress as observed in many cancer cells [39].

Assessment of carcinogenic potential of chemical agents to which human beings are exposed is clearly of prime importance. Chemical carcinogens are divided into genotoxic and non-genotoxic classes on the basis of their ability to react with DNA and form adducts. The mechanisms of carcinogenesis induced by exposure to genotoxic agents are suggested to be related to the damage of target cells DNA, formation of DNA adducts, errors in DNA repair and fixation of mutations induced by metabolically activated ultimate carcinogens, leading to activation of cell proliferation and formation of preneoplastic lesions and tumors (benign and malignant) [40]. However, many chemicals producing tumors in laboratory animals were shown to act by epigenetic mechanisms that do not involve DNA attack or hereditable genetic alteration [41]. It was accepted that the indirect nature of the mechanisms involved means that prolonged exposure to high levels of chemicals is necessary for the production of tumors [42]. With such non-genotoxic carcinogens, theoretically cancer would not occur at exposure below a threshold at which the relevant cellular effect is not operative.

It has been recently discovered that body respond differentially to the high and low doses of chemical carcinogens. Interestingly, in the field of carcinogenesis, a lot of attention has been attracted to the phenomenon of hormesis, a biphasic dose-response relationship, in which a toxicant exerts opposite effects dependent on the dose. There is considerable experimental evidence that non-genotoxic agents exert hormetic effects. Thus, several studies in experimental animals have demonstrated that low doses of non-genotoxic carcinogens may inhibit hepatocarcinogenesis. The possible mechanisms of carcinogenic hormesis with cytochrome P_450_ inducers, such as PB [43], α-BHC [44,45] and DDT [46] included suppression of generation of oxidative DNA base modifications in terms of 8-OHdG in the rat liver. Furthermore, epigenetic processes differentially can be affected by agents that impinge on oxidative stress, DNA repair, apoptosis, cell proliferation, intracellular communication and cell signaling. Non-genotoxic carcinogens may target nuclear receptors, cause aberrant DNA methylation at the genomic level and induce post-translational modifications at the protein level, thereby impacting on the stability or activity of key regulatory proteins, including oncoproteins and tumor suppressor proteins. Via multiple epigenetic lesions, non-genotoxic carcinogens can elicit a variety of changes contributing to cellular carcinogenesis.

## 3. Hormesis in Carcinogenicity of Non-Genotoxic Carcinogens

### 3.1. Carcinogenicity of Phenobarbital (PB)

Non-genotoxic hepatocarcinogen, PB exerts promoting effects on hepatocarcinogenesis after suitable initiation in rats [47], and enhances the proliferation of carcinogen-exposed hepatocytes *in vitro* [48]. PB chronic administration to mice and rats results in liver carcinogenicity [49,50]. Inhibition of cell proliferation, DNA synthesis and increased TGF-β_1_ concentrations have all been observed in the liver tissue depending on the time point [51,52]. On the other hand, growth of preneoplastic lesions induced by PB might predominantly be attributable to an inhibition of programmed cell death/apoptosis or to induction of ROS, oxidative DNA damage and genetic alterations by spontaneous errors in DNA replication and repair [53,54].

**Figure 1:**
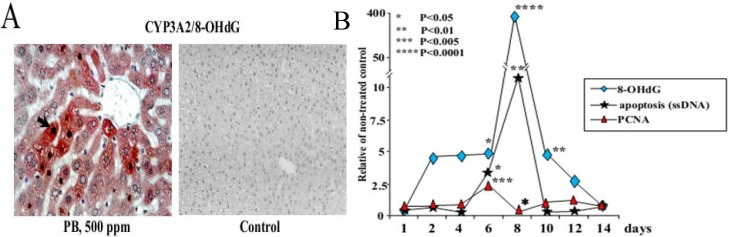
(**A**) Representative microphotograph of double staining for 8-OHdG (black/brown) and CYP3A2 (red) in the livers of rats treated with 500 ppm PB for 8 days (×400). Note, the increase of 8-OHdG in the nuclei of pericentrally localized hepatocytes most strongly stained for CYP3A2; (**B**) Alteration to 8-OHdG, apoptosis and cell proliferation in the liver of rats treated with 500 ppm PB.

Previously, to evaluate the risk of exposure to non-genotoxic chemicals and elucidate mechanisms underlying their promoting activity on rat liver carcinogenesis the formation of 8-OHdG, P_450_ and hydroxyl radicals induction, DNA repair, and alteration to apoptosis and cellular proliferation in the rat liver were investigated during short-term administration of PB at a carcinogenic dose of 500 ppm [36]. Significant elevation of hydroxyl radical levels by day 4 of PB exposure accompanied the accumulation of 8-OHdG in the nucleus and CYP2B1/2 and CYP3A2 in the cytoplasm of hepatocytes (Table 1 and Figure 1A). 

**Table 1:** Key *in vivo* findings observed with several non-genotoxic and genotoxic chemical carcinogens.

Chemical	Key *in vivo* findings
PB	Induction of P_450_ isoenzymes (e.g., CYP2B and CYP3A), 8-OHdG, cell cycle arrest and apoptosis in the rat liver (short-term study) Promotion of hepatocarcinogenesis in rats at high doses (60–500 ppm) and inhibition at low doses (1 and 2 ppm) (medium-term rat liver bioassay, 2-step carcinogenicity test)
α-BHC	Promotion of hepatocarcinogenesis in rats at high doses (0.5 to 500 ppm) and inhibition at low doses (0.01 and 0.1 ppm) (medium-term rat liver bioassay, 2-step carcinogenicity test)
DDT	Promotion of hepatocarcinogenesis in rats at high doses (20–500 ppm) and inhibition at low doses (0.005 and 0.01 ppm) (medium-term rat liver bioassay, 2-step carcinogenicity test)
ETBE	Induction of P_450_ (e.g., CYP2B, CYP3A, CYP2E1), 8-OHdG, cell cycle arrest and apoptosis in the rat liver (short-term study) Tumorigenicity in the rat liver (2-year carcinogenicity test) and promotion of hepatocarcinogenesis in rats (multi-organ carcinogenesis bioassay)
Organic arsenicals	
MMA^V^	Promotion of preneoplastic lesions development in the liver (medium-term rat liver bioassay)
DMA^V^	Induction of P_450_ isoenzymes (e.g., CYP2B1, CYP3A1 and CYP1A2), phase II metabolizing enzymes, 8-OHdG, cell cycle arrest and apoptosis in the rat liver (short-term study) Promotion of carcinogenesis in the rat urinary bladder, kidney, liver and thyroid gland (multi-organ carcinogenesis bioassay, medium-term rat liver bioassay) Carcinogenicity in the rat bladder (2-year carcinogenicity test) Carcinogenicity in the lung of *Mmh/Ogg1*-deficient mice (long-term carcinogenicity test)
TMAO^V^	Induction of P_450_ isoenzymes (e.g., CYP2B1 and CYP1A2), phase II metabolizing enzymes, 8-OHdG, cell cycle arrest and apoptosis in the rat liver (short-term study) Promotion of preneoplastic lesions in the rat liver (medium-term rat liver bioassay) Tumorigenicity in the rat liver (2-year carcinogenicity test)
MeIQx	Induction of GST-P positive foci and liver tumors (100 to 400 ppm), formation of MeIQx-DNA adducts, elevation of 8-OHdG, *lacI* and H-*ras* mutation levels (long-term carcinogenicity test)
KBrO_3_	Increase of *lacI* mutations, 8-OHdG levels, and promotion of carcinogenesis in the rat kidney (2-step carcinogenicity test)

Conspicuous elevation of 8-OHdG and apoptosis in the liver tissue were associated with reduction of proliferating cell nuclear antigen (PCNA) index after 8 days of PB application (Figure 1B). Thereafter 8-OHdG levels decreased with increase in mRNA expression for the enzyme responsible for the repair of 8-OHdG lesions, oxoguanine glycosylase 1 (Ogg1). Induction of cyclin D1 (CD1) and p21^WAF1/Cip1^ mRNA expression on days 4 and 6, respectively, preceded marked elevation of PCNA and apoptotic indices. Authors suggested that non-genotoxic chemicals might induce reversible alteration to nuclear 8-OHdG in the rat liver after several days of continuous application [36]. Increased 8-OHdG formation is likely to be the result of developing oxidative stress or apoptotic degradation of DNA and coordinated with enhanced expression of CD1 mRNA and cell proliferation, subsequent increase of p21^WAF1/Cip1^ mRNA expression, cell cycle arrest and apoptosis, while activation of 8-OHdG repair mechanisms contributes to protection of tissue against ROS-induced DNA damage and cell death.

Especial attention has been recently devoted to the carcinogenicity of low doses of PB. To determine the threshold level for its hepatopromoting effects, firstly, the dose dependence was investigated using a rat liver medium-term bioassay (Ito test) (Table 1) [55,56]. When the chemical was administered to 6 week-old F344 rats in a wide range of doses of 0.01 (the lowest) to 500 ppm (the highest) in the diet for 6 weeks, with 2/3 partial hepatectomy after a single intraperitoneal injection of DEN at 200 mg/kg in serial experiments, the end point marker of hepatocarcinogenesis in rats, glutathione S-transferase placental form (GST-P) positive foci, were found to be increased dose-dependently in rats given 60–500 ppm. However, with doses in the range of 1–7.5 ppm, decrease was evident as compared to the control group, being statistically significant at 1 and 2 ppm. It was concluded that PB exerted hormetic effect in the rat liver, indicating the existence of a threshold for its carcinogenicity, which is likely to be related to suppression of CYP3A2 protein expression by low doses of the chemical.

**Figure 2:**
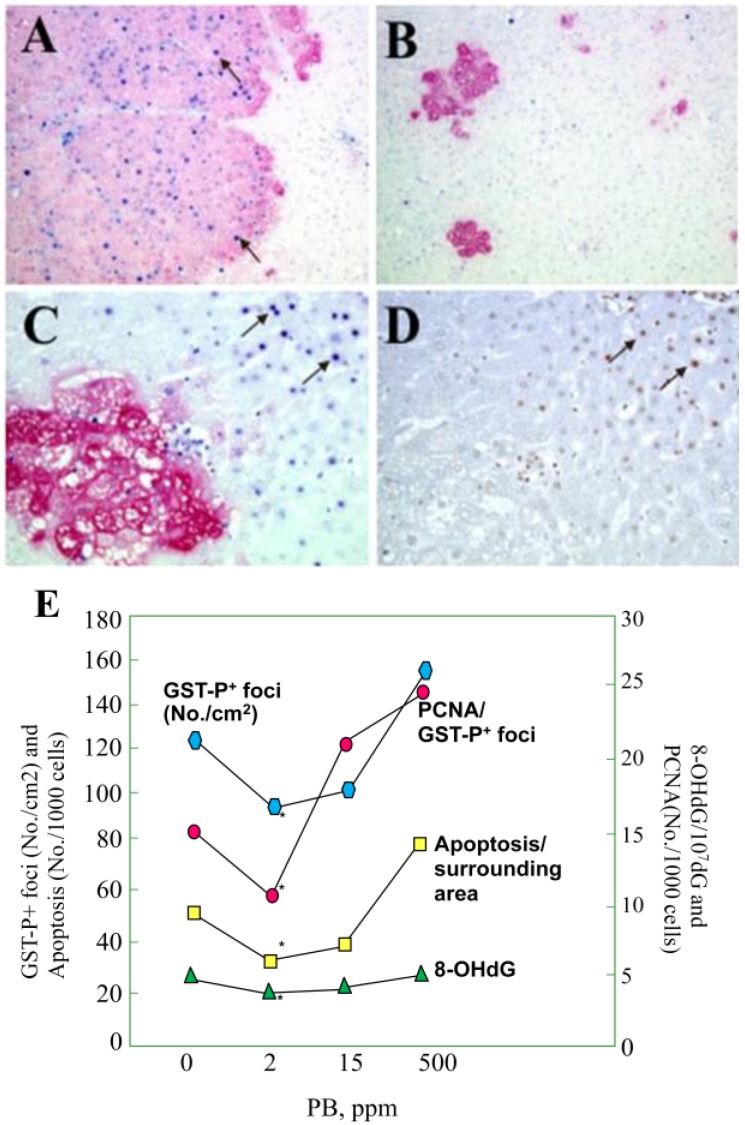
Double immunohistochemistry for GST-P (red) and PCNA (blue), GST-P (red) and apoptosis (ssDNA) (blue) and immunohistochemical assessment of 8-OHdG (black/brown) in the livers of F344 rats treated with PB for 10 weeks after DEN initiation. (**A** and **B**) GST-P and PCNA after PB at 500 ppm (×100); and 2 ppm (×100), respectively; (**C**) GST-P and apoptosis after PB at 500 ppm (×200); (**D**) 8-OHdG immunohistochemistry in the liver of the rat treated with PB at 500 ppm (×200). Note, that *C* and *D* are serial liver sections and the good concordance between 8-OHdG and ssDNA stainings; (**E**) Co-ordinated change in GST-P positive foci numbers and PCNA indices, for apoptosis and 8-OHdG levels in background liver parenchyma after DEN then PB for 10 weeks. Note the inhibitory effects of PB at the dose of 2 ppm and elevation at 500 ppm.

The hormetic influence of PB was further clarified in an *in vivo* experiment in which male F344 rats were treated with the chemical at doses of 0, 2, 15 and 500 ppm in diet for 10 or 33 weeks after initiation of hepatocarcinogenesis with DEN (Table 1) [43]. After 10 and 33 weeks of administration, formation of liver tumors and GST-P positive foci was inhibited at 2 ppm. Significant reduction in the multiplicity of total tumors, in particular, hepatocellular carcinomas (HCCs), and a tendency for decreased incidences of HCCs and hepatocellular adenomas at 2 ppm was demonstrated. However, 500 ppm PB treatment resulted in strong elevation of HCC and total tumor multiplicities. Interestingly, the mechanism of hepatocarcinogenicity inhibition at 2 ppm was considered to be due to the suppression of 8-OHdG generation and cellular proliferation within areas of GST-P positive foci, as well as apoptosis, in background liver parenchyma (Figure 2A,B). Furthermore, the decrease of 8-OHdG levels induced by PB at low dose was possibly a result of elevated expression of Ogg1.

### 3.2. Carcinogenicity of α-Benzene Hexachloride (α-BHC)

A major organochlorine byproduct in the manufacture of lindane (γ-BHC), α-BHC, has been used in admixtures with lindane for agricultural purposes. Among the eight isomers of BHC, the α-isomer has been categorized as a non-genotoxic carcinogen as it has been shown to have no mutagenicity in the Ames test but to induce liver tumors in rodents after high dose administration. 2,4,6-Trichlorophenol is the major metabolite in α-BHC metabolism by the cytochrome P_450_ oxidoreductase system. Removable chlorine atoms might react with hydrogen peroxide to produce hypochlorous radicals binding to DNA and formation of chlorinated DNA adducts, like 8-chloro-2-deoxyguanosine, 5-chloro-2-deoxycytidine and 8-chloro-2-deoxyadenosine after dechlorination and dehydrochlorination of α-BHC [57,58]. Long-term treatment with α-BHC, but not β- and γ-BHC, at high doses (500 or 1,000 ppm) has been revealed to induce hyperplastic nodules and liver carcinomas in rats and mice [59,60]. Furthermore, several toxicological studies found that α-, β-, and γ-BHC are potent inducers of hepatic monooxygenases in rats [61], in addition to causing liver enlargement [62]. 

The dose dependence of α-BHC promoting effects regarding rat hepatocarcinogenesis was first investigated by Masuda *et al*. [44] in a medium-term rat liver bioassay (Table 1). When F344 male rats were given α-BHC at a wide range of doses from 0.01 to 500 ppm in their diet for 6 weeks after a single intraperitoneal injection of DEN, quantitative values for numbers and areas of GST-P positive foci were dose-dependently increased at 0.5 to 500 ppm. On the contrary, a tendency for a decrease was observed with 0.01 and 0.1 ppm α-BHC. In line with PB data, CYP3A2 protein levels and activities showed a good correlation with the numbers and areas of GST-P positive foci. Supportive evidence for hormesis in the promotion of rat hepatocarcinogenesis by α-BHC was provided and the mechanism was suggested to be related to suppression of CYP3A2 protein expression by low doses of the chemical [44]. 

The possibility of a hormetic effect of α-BHC regarding formation of liver preneoplastic lesions and tumors *in vivo*, was further examined in F344 rats at doses from 0.01 to 500 ppm in the diet for 10 and 36 weeks after initiation of hepatocarcinogenesis with DEN (Table 1) ([63,64]). The formation of GST-P positive foci was promoted by α-BHC treatment at the dose of 500 ppm, however, both their numbers and areas were found to be significantly reduced with 0.05 ppm. Furthermore, incidences and multiplicities of liver tumors were found increased in a dose-dependent manner by α-BHC at doses of 0.5–500 ppm, while a tendency for decrease in their values was found in the low dose 0.01 and 0.1 ppm groups, similar to the case with rat liver preneoplastic lesions. At week 10, dose response curves of cytochrome P_450_ total content, NADPH-cytochrome P_450_ reductase activity and 8-OHdG formation reflected those of GST-P positive foci. Similar to the PB hepatocarcinogenicity case, α-BHC at low dose also tended to up-regulate Ogg1 mRNA expression. As for PB, 500 ppm α-BHC treatment led to increase in cell proliferation within areas of GST-P positive foci but decreased their values at low doses. The response curves for catalytic activity, protein levels and mRNA expression of CYP2B1 and CYP3A2 exhibited thresholds, but CYP2C11 activity exhibited an inverted J-shape. This major constitutive male-specific P_450_ isoform, CYP2C11 was found to be up-regulated by a low dose of α-BHC treatment at the transcriptional level and with regard to catalytic activity. Thus, CYP2C11 was suggested to take part in detoxification while CYP2B1 and CYP3A2 isoenzymes and considered to participate in bioactivation of α-BHC and increase of its hepatotoxicity, given the correlation with GST-P positive foci and 8-OHdG. The non-linear threshold dose response observed at low doses with respect of CYP2B1 and CYP3A2 can be deemed a result of a multi-step process “turning on” orphan nuclear receptors, CAR and PXR, which are known to regulate CYP2B1 and CYP3A2 transcription [65,66]. From these results α-BHC was concluded to exert hormetic effect regarding its hepatocarcinogenicity by mechanisms involving induction of detoxifying enzymes at low dose, as well as influencing free radical production and oxidative stress, and consequently pathological change in the liver.

### 3.3. Carcinogenicity of 1,1-bis(p-Chlorophenyl)-2,2,2-trichloroethane (DDT)

Inhibitory effects on development of GST-P positive foci were also noted with low doses of the organochlorine insecticide and a non-genotoxic carcinogen, DDT. Firstly, 21-day-old F344 rats, received DDT at doses from 0.005 to 500 ppm in their diet for 16 weeks (Table 1) [67]. Secondly, Kushida *et al*. [46] re-examined the possibility of hormesis after DDT administration to F344 rats for 11 and 43 weeks after the initiation of hepatocarcinogenesis with DEN (Table 1). In both studies, the doses of 20 ppm and above were associated with dose-dependent induction of GST-P positive foci in the liver. On the contrary, in the low doses of 0.005 and 0.01 ppm groups, a tendency for decrease in values below the control level was observed. Moreover, histopathological analysis of liver nodules revealed a tendency for decrease in the incidence and multiplicity of HCCs in the low dose groups as compared to the DEN initiation controls. Interestingly, the correlation was found with PB and α-BHC, as the numbers and areas of GST-P positive foci in the low dose DDT groups was correlated with a tendency for decrease in the CYP3A2 protein level as well as induction of IL-1 receptor type I (IL-IRI) and TNF-α receptor type I, whose ligands have roles in downregulating CYP3A2 and influencing cellular proliferation or apoptosis [67]. 

It was found that within GST-P positive areas, cell proliferation was slightly lower in the 0.005 ppm DDT dose group as compared to DEN initiation control [67]. As observed in experiments with PB and α-BHC, CYP2B1/2 and CYP3A2 protein levels in the liver microsomal fraction were significantly elevated by high doses of DDT. Furthermore, in line with previous results, 8-OHdG formation was significantly suppressed by low dose of DDT, presumably related to effective DNA repair, which may exceed formation of adducts. Oxidative stress was suggested to be decreased in the low dose group because of the lowered CYP3A2 expression and 8-OHdG formation balanced through elimination by Ogg1 [46,67]. Furthermore, in the low DDT dose group, mRNA and protein expressions of connexin 32 (Cx32) were found to be elevated [46]. Previously, numerous studies reported that high doses of DDT and other non-genotoxic carcinogens inhibit Cx32, resulting in the loss of function of gap junction intracellular communication and release of potentially initiated cells from growth constraints imposed by normal neighboring cells, leading to clonal expansion and ultimately tumor formation and progression [68,69].

## 4. Carcinogenicity of Ethyl *Tertiary*-Butyl Ether (ETBE)

ETBE is a well-known chemical and gasoline oxygenate synthesized from bioethanol and isobutene which is used as a gasoline blending component in order to reduce the exhaust emissions such as carbon monoxide, unburned hydrocarbons, polycyclic aromatics and oxides of nitrogen. Nevertheless, humans are at risk of exposure to oxygenates not only by inhalation while fueling automobiles but also orally when drinking contaminated water [70,71]. ETBE has been demonstrated to be non-genotoxic in several test systems including gene mutation tests using Chinese hamster ovary (CHO) cells and *Salmonella typhimurium* strain, chromosomal aberration test with CHO cells, and *in vivo* micronucleus test with bone marrow cells of mice orally treated with ETBE and mice exposed to ETBE by inhalation [71].

Importantly, ETBE-administered to male F344 rats by inhalation at a dose of 5,000 ppm for 2-years has been recently shown to induce the development of liver preneoplastic lesions (eosinophilic and basophilic foci) and hepatocellular adenomas (Table 1) [72]. Furthermore, in a multi-organ carcinogenesis bioassay, 1,000 mg/kg b.w./day ETBE by gavage was found to promote hepatocarcinogenesis in male F344 rats [73]. Recently, for elucidation of possible mode of action (MOA) and human relevance of hepatotumorigenicity in rats for ETBE, a short-term study was performed in F344 rats which were administered ETBE at doses of 0, 150 and 1,000 mg/kg body weight twice a day by gavage for 1 and 2 weeks [35]. Significant increase of P_450_ total content and hydroxyl radical levels by both doses of ETBE treatments at weeks 1 and 2, and 8-OHdG formation at week 2, accompanied accumulation of CYP2B1/2, CYP3A1/2 and CYP2C6 and induction of apoptosis and cell cycle arrest in hepatocytes, respectively. The similarities were found with PB MOA. Up-regulation of CYP2E1 and CYP1A1 at week 1 and 2, and peroxisome proliferation at week 2 was found in high dose ETBE group. Results of proteome analysis predicted activation of upstream regulators of gene expression altered by ETBE including CAR, PXR and PPAR nuclear receptors. The results demonstrated that short-term exposure to the non-genotoxic chemical, ETBE administered to rats by gavage activated CAR and PXR nuclear receptors in the livers similar to the mechanism possessed by the non-genotoxic chemical carcinogen, PB, and furthermore specifically activated PPARs, thus, leading to conspicuous elevation of 8-OHdG formation, cell cycle arrest and apoptosis due to the development of oxidative stress, activation of fatty acid metabolism in mitochondria and peroxisome proliferation in hepatocytes [35].

## 5. Carcinogenicity of Genotoxic Carcinogens and Involvement of Oxidative Stress

### 5.1. Arsenic Carcinogenicity

Inorganic arsenicals, trivalent arsenite (As^3+^) and pentavalent arsenate (As^5+^), are naturally occurring and ubiquitously present in the environment. Epidemiological data have shown that long-term exposure of humans to inorganic arsenic compounds is associated with liver injury, peripheral neuropathy, and an increased incidence of cancers of the lung, skin, urinary bladder, and liver [74,75]. Organic arsenicals monomethylarsonic acid (MMA^V^), dimethylarsinic acid (DMA^V^) and trimethylarsine oxide (TMAO^V^) are the primary metabolites of inorganic arsenic formed by methylation, the major pathway of inorganic arsenic biotransformation and detoxification, which requires reduced cellular glutathione. 

Recent data from biochemical and carcinogenic studies of DMA^V^ suggest that the methylation of arsenic may result in activation instead of detoxification [76]. In a rat multiorgan carcinogenesis bioassay based on the two-stage model of carcinogenesis, DMA^V^ in drinking water has been reported to promote carcinogenesis in the rat urinary bladder, kidney, liver and thyroid gland [77,78], and exhibit carcinogenicity in the urinary bladder of rats in a two-year carcinogenicity test (Table 1) [79]. MMA^V^, DMA^V^ and TMAO^V^ were also shown to enhance the formation of preneoplastic lesions in the liver, when administered to F344 rats in a drinking water in a medium-term bioassay (Table 1) [80]. Furthermore, TMAO^V^ have been shown to exert tumorigenic potential in the liver, when administered to F344 rats in the drinking water in a 2-year study (Table 1) [81]. Arsenic carcinogenicity is considered to involve complex mechanisms, which could involve more than one MOA. Oxidative stress, stimulation of cell proliferation and induction of chromosomal abnormalities are considered to be the three main possibilities of arsenic carcinogenic action [82]. Previously, in long-term carcinogenicity tests and medium-term liver bioassay, increased formation of 8-OHdG and stimulation of cell proliferation were implicated as possible mechanisms of arsenical carcinogenicity in the liver, urinary bladder and kidney of rats [77,79,80]. In mice, DMA^V^-induced lung-specific DNA damage can be attributed to free radicals, particularly dimethylarsenic peroxyl radical ((CH_3_)_2_AsOO^·^), arising from the reaction of DMA^V^ with molecular oxygen [83]. 

**Figure 3:**
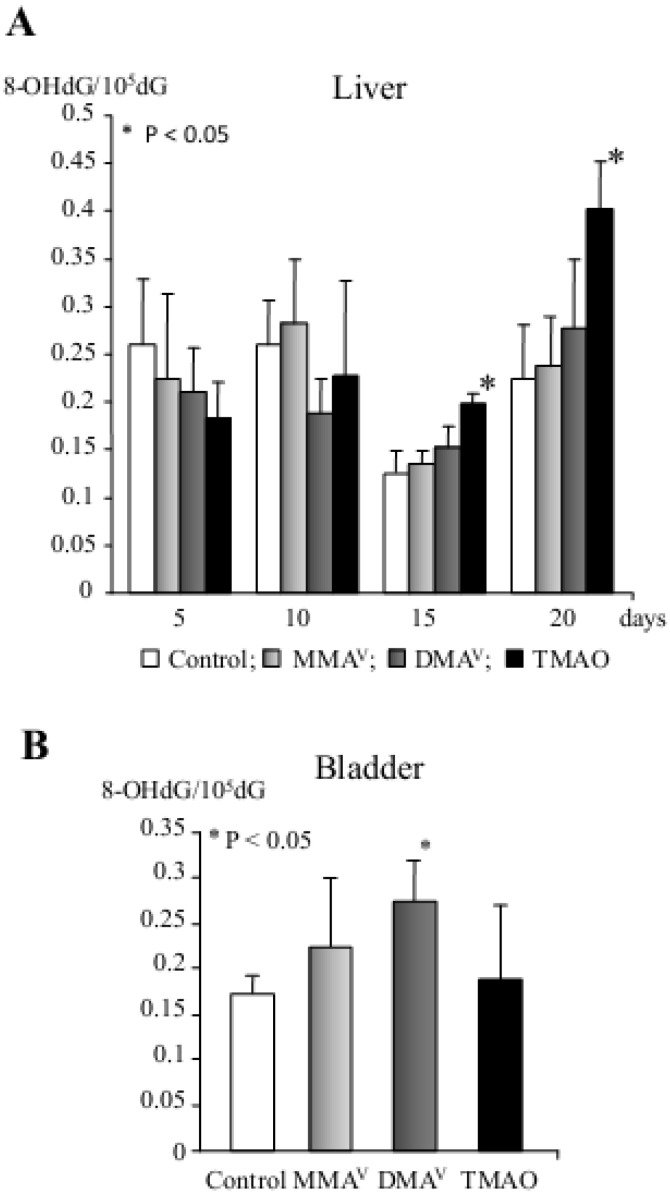
8-OHdG formation in the rat liver (**A**) and bladder (**B**, 20th day only) after MMA^V^, DMA^V^ and TMAO administration.

To clarify the mechanisms of organic arsenicals’ carcinogenicity and to compare biological responses in the liver and bladder, a short-term study was performed in male F344 rats which were sequentially treated for 5, 10, 15, 20 days with MMA^V^, DMA^V^ and TMAO^V^ in their drinking water at a dose of 200 ppm (Table 1) [84]. P_450_ total content and generation of hydroxyl radicals were significantly increased in the rat livers from 10 and 15 days of treatment with arsenicals, respectively, with the highest levels induced by TMAO^V^. Similarly, elevation of 8-OHdG formation was found in the DNA with significant increase by TMAO^V^ treatment in the liver at days 15 and 20, and DMA^V^ in the bladder after 20 days treatment (Figure 3). In addition, cell proliferation and apoptosis indices were significantly increased by TMAO^V^ in the liver and by DMA^V^ in the bladder of rats. These events were accompanied by differential up-regulation of phase I (CYP2B1 and CYP1A2 in the liver and CYP2B1, CYP3A1 and CYP1A2 in the bladder) and phase II metabolizing enzymes, cyclins D1 and E, PCNA, caspase 3 and FasL. The results indicated that early elevation of 8-OHdG and cell proliferation via generation of oxidative stress by TMAO^V^ and DMA^V^ contributes to their carcinogenicity in the rat liver and bladder [84]. These results were further supported by another study, in which DMA^V^ was shown to exert carcinogenicity in the lungs of homozygous *Mmh/Ogg1*-deficient mice defective in repair of oxidative DNA base modifications [85]. These data indicated that significant persistent elevation of 8-OHdG, with induction of *Pola1*, *Cyp7b1*, *Ndfua3*, *Mmp13* and *Hspb1* genes expression induced by DMA^V^ with high background levels of cell proliferation might be responsible for DMA^V^ carcinogenicity in the *Ogg1*^−/−^ mutant mouse lung.

### 5.2. Carcinogenicity of 2-Amino-3,8-dimethylimidazo[4,5-f]quinoxaline (MeIQx)

**Figure 4:**
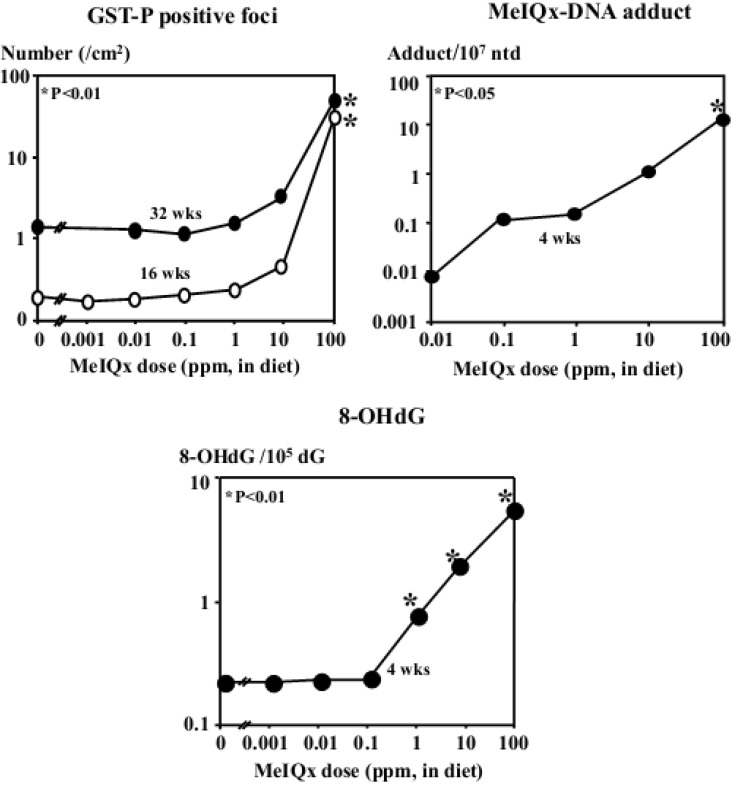
Hepatocarcinogenicity of MeIQx at low doses in rats.

**Figure 5:**
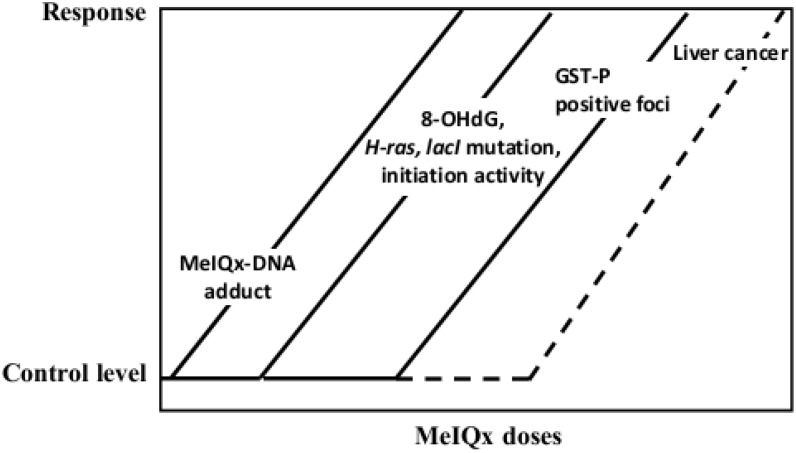
Risk of liver cancer: Reaction curves of the carcinogenicity markers dependent on the dose of MeIQx.

A genotoxic carcinogen, MeIQx is a heterocyclic amine contained in fried meat and fish, and 100 to 400 ppm doses of MeIQx were found to be carcinogenic in the rat liver (Table 1) [86].To investigate the effect of MeIQx exposure at low doses, 1145, 21-day-old male F344 rats were administered MeIQx in the diet at doses of 0, 0.001, 0.01, 0.1, 10 ppm (low dose groups) or 100 ppm (high dose group) for 16 and 32 weeks [87]. The total numbers and areas of GST-P positive foci were not changed in the 0.001–1 ppm MeIQx groups, however, at 10 ppm there was an indication for an increase and at 100 ppm a significant elevation was noted as compared to non-treated controls, both in 16 and 32-week studies (Figure 4). Furthermore, after 4 and 16 weeks of administration, the formation of MeIQx-DNA adducts was dose-dependently induced. 8-OHdG formation was elevated at MeIQx doses higher than 1 ppm. Moreover, the mutation level of H-*ras*, which role in rat hepatocarcinogenesis is still unclear, was significantly increased in livers of rats treated with MeIQx at doses higher than 1 ppm [88]. Interestingly, in a Big Blue transgenic rat mutagenesis assay, MeIQx at doses of 1 ppm and less was found not to induce *lacI* mutations in the liver, loosely correlating with non-induction of GST-P positive foci [89]. On the other hand, the MeIQx dose at which significant increase of *in vivo* mutagenicity was noted, appeared to be lower than the dose inducing formation of GST-P positive foci (Figure 5). Increase of carcinogen-DNA adducts, 8-OHdG levels, *in vivo* mutagenicity, H*-ras* mutations, development of GST-P positive foci and lastly, liver tumors appeared to be the chain of sequential events, dependent on the dose of carcinogen, indicating the existence of threshold, at least a “practical” threshold for carcinogenicity of genotoxic carcinogens such as MeIQx.

### 5.3. Carcinogenicity of Potassium Bromate (KBrO_3_)

KBrO_3_, known as a pollutant of tap water and as a food additive, was shown to exert carcinogenicity in the rat kidney (Table 1) [90]. Furthermore, KBrO_3_ was reported to belong to the class of the DNA non-reactive genotoxic carcinogens [90]. To investigate the mutagenicity in the rat kidney, it was administered to Big Blue rats in their drinking water at doses of 0.02 to 500 ppm for 16 weeks [91]. The significant increase of *lacI* mutations was found at a dose of 500 ppm, however, no mutagenicity was observed for doses lower than 125 ppm. At a dose of 500 ppm, KBrO_3_ was found to induce oxidative DNA damage-specific mutations (GC to TA transversions). Furthermore, the significant increase of 8-OHdG level in the kidney was detected only at a dose of 500 ppm. In another study, for the examination of KBrO_3_ carcinogenic potential in the kidney, male Wistar rats were treated with *N*-ethyl-*N*-hydroxyethylnitrosamine for initiation of kidney carcinogenesis, and thereafter administered KBrO_3_ over wide dose range [92]. Increase in induction of kidney tumors was obvious in the highest dose group (the dose was changed during the experiment from 500 to 250 ppm due to the developed toxicity), but not for the dose of 125 ppm or lower. Furthermore, formation of 8-OHdG in rat kidneys was significantly elevated by KBrO_3_ at doses of 125 ppm and above but not 30 ppm and below. The results indicated that only high doses of KBrO_3_ induced oxidative DNA damage and exerted promotion effects on development of EHEN-initiated kidney lesions. 

## 6. Induction of ROS, Cell Proliferation, Apoptosis and DNA Repair

**Figure 6:**
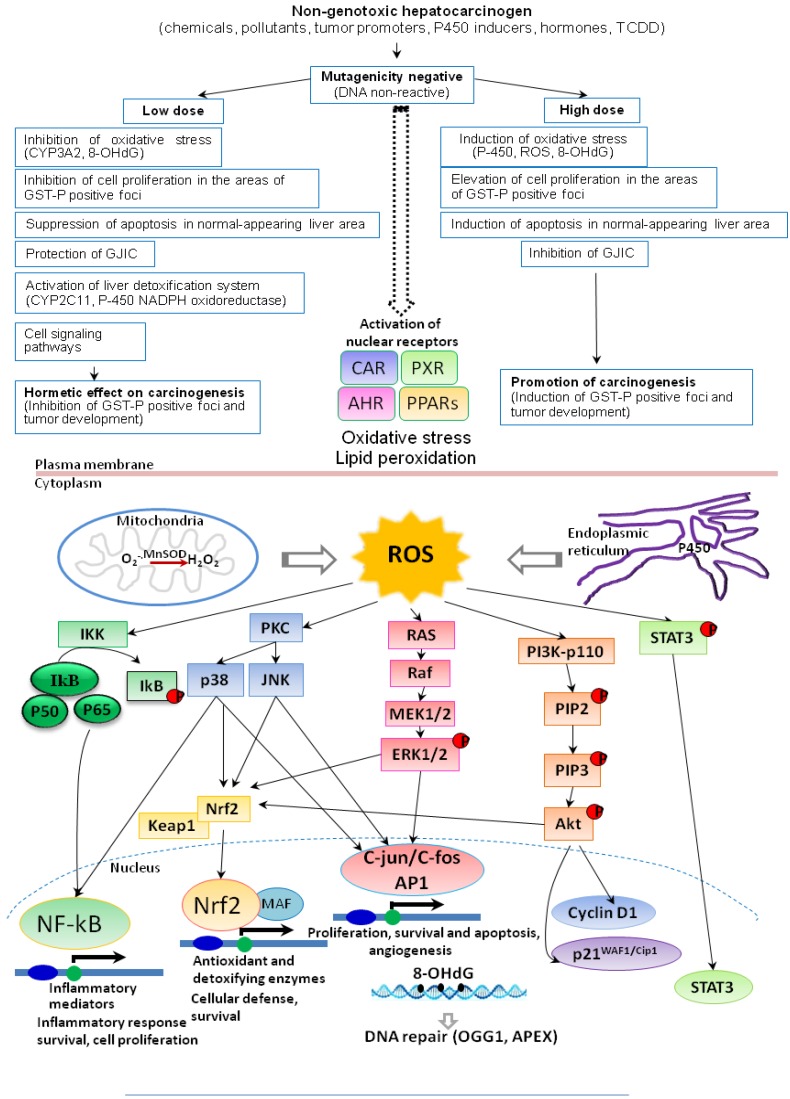
Flow scheme toward dose-effect relations and mechanisms of action of non-genotoxic chemical carcinogens.

Exposure to different chemical carcinogens for which hormetic effects are proposed frequently leads to induction of cytochrome P_450_ species and formation of ROS, thus producing oxidative stress (Figure 6). However, many aspects have already been elucidated, indicating that at low levels of ROS, adaptive responses, repair and antioxidative defenses are strengthened, whereas at high levels they may be overwhelmed. Whether induction of a detoxifying enzyme qualifies as a basis for a practical threshold depends on the speed and capacity of removal of the reactive species from the system compared with the speed of the translocation of the reactive species from the site of its generation to the nucleus and reaction with the DNA. 

Induction of ROS has been observed to alter cell proliferation and apoptosis (Figure 6). While marked increase in oxygen radicals in the rat liver in cases of non-genotoxic carcinogens, PB, α-BHC and DDT at high dose, for example, leads to elevation of cell proliferation in areas of GST-P positive foci, cell proliferation rates at low doses were found to be decreased in the foci [43]. Suppression of liver nuclear DNA 8-OHdG formation at low dose may be associated with reduction of cell proliferation within GST-P positive foci. Furthermore, apoptosis, significantly induced by high dose administration in liver tissue surrounding GST-P foci, was suppressed in the low dose groups, with strong similarity to the pattern observed for 8-OHdG. Apoptosis of normal-appearing liver tissue has been proposed as one factor regulating the size of foci, as enlargement of GST-P positive foci presumably requires regenerative stimuli. In the rat liver treated with PB, the results of cDNA microarray analysis indicated low dose to specifically enhance mRNA expression for glutamic acid decarboxylase (GAD65), an enzyme involved in the synthesis of γ-aminobutyric acid (GABA), while suppressing expression of MAP kinase p38, JNK1, 2 and other intracellular kinases [43] (Figure 6). Recently, a negative correlation between the expression of GABA-A receptors in hepatocytes and thymidine incorporation in liver specimens was reported, albeit without evidence of a causal relationship, and the GABA-B receptor subtype is known to be involved in hepatocyte DNA synthesis and mediation of growth stimulation [93,94]. Thus, the suppression of gene expression of signal transduction modulators, such as MAP kinase p38, JNK1, 2 and other intracellular kinases might be a factor related to the inhibitory effect of PB at low dose on cell proliferation.

The fact that DNA repair protects cells from fixation of DNA damage in the newly synthesized DNA strand as heritable mutations, means that outcome of exposure to carcinogens is dependent on the race between repair and proliferation-dependent DNA synthesis. The combination of elevated repair and decreased cell division may be more than compensation for deleterious influence. Application of high doses of the same substance may result in tumor induction because of cell cycle acceleration due to cytotoxicity and regenerative cell proliferation which represent the key parameters concerning threshold mechanisms, although apoptosis also contributes. This would be important for non-genotoxic carcinogens. Furthermore, it should be borne in mind that apoptosis and the control of neoplastically transformed cells by the immune system may be additional factors influencing the shape of the dose-response curve in carcinogenicity.

## 7. Conclusions

Recent data on the effects of non-genotoxic carcinogens such as PB, α-BHC, and DDT indicate the existence of hormesis which implies the maintenance of homeostasis, with adaptive responses involving alteration to formation of oxidative stress, DNA damage and repair, apoptosis, cell proliferation and signaling, and cell-cell communication. The findings have broad implications for the present concept of threshold in non-genotoxic carcinogens response and therefore, for cancer risk assessment of medicines, agrichemicals, food additives and so on.

In case of genotoxic carcinogens, increase of DNA adducts at low doses preceded the elevation of 8-OHdG at somewhat higher doses, related to rise in gene mutations, and lastly by formation ofpreneoplastic lesions. These data point the primary importance of formation of DNA adducts by genotoxic chemicals, which contributes to the initiation stage of carcinogenesis. The probable secondary mechanism for their carcinogenicities involves the formation of oxidative stress which is likely to be implicated in promotion and progression stages. In addition, the possibility of existence of threshold for the carcinogenicities of genotoxic carcinogens is the next question of interest. 
